# Optimizing Diagnostic Performance of ChatGPT: The Impact of Prompt Engineering on Thoracic Radiology Cases

**DOI:** 10.7759/cureus.60009

**Published:** 2024-05-09

**Authors:** Turay Cesur, Yasin Celal Güneş

**Affiliations:** 1 Radiology, Ankara Mamak State Hospital, Ankara, TUR; 2 Radiology, Kırıkkale Yuksek Ihtisas Hospital, Ankara, TUR

**Keywords:** prompt engineering, radiology, large language models, gpt-4, chat generative pre-trained transformer (chatgpt)

## Abstract

Background

Recent studies have highlighted the diagnostic performance of ChatGPT 3.5 and GPT-4 in a text-based format, demonstrating their radiological knowledge across different areas. Our objective is to investigate the impact of prompt engineering on the diagnostic performance of ChatGPT 3.5 and GPT-4 in diagnosing thoracic radiology cases, highlighting how the complexity of prompts influences model performance.

Methodology

We conducted a retrospective cross-sectional study using 124 publicly available *Case of the Month* examples from the *Thoracic Society of Radiology *website. We initially input the cases into the ChatGPT versions without prompting. Then, we employed five different prompts, ranging from basic task-oriented to complex role-specific formulations to measure the diagnostic accuracy of ChatGPT versions. The differential diagnosis lists generated by the models were compared against the radiological diagnoses listed on the Thoracic Society of Radiology website, with a scoring system in place to comprehensively assess the accuracy. Diagnostic accuracy and differential diagnosis scores were analyzed using the McNemar, Chi-square, Kruskal-Wallis, and Mann-Whitney U tests.

Results

Without any prompts, ChatGPT 3.5's accuracy was 25% (31/124), which increased to 56.5% (70/124) with the most complex prompt (*P *< 0.001). GPT-4 showed a high baseline accuracy at 53.2% (66/124) without prompting. This accuracy increased to 59.7% (74/124) with complex prompts (*P *= 0.09). Notably, there was no statistical difference in peak performance between ChatGPT 3.5 (70/124) and GPT-4 (74/124) (*P *= 0.55).

Conclusions

This study emphasizes the critical influence of prompt engineering on enhancing the diagnostic performance of ChatGPT versions, especially ChatGPT 3.5.

## Introduction

Large language models (LLMs) represent a paradigm shift in the field of artificial intelligence (AI), offering remarkable capabilities for understanding and generating human-like text based on vast datasets [1,2]. The capabilities of LLMs, especially OpenAI’s foundational LLM, ChatGPT 3.5, have attracted enormous attention in healthcare research [1]. This attention is significant in the realm of medical sciences, where AI's potential to augment scholarly work, diagnostic accuracy, and clinical decision-making is being increasingly recognized [3].

In radiology, the application of LLMs promises to revolutionize diagnostic methodologies. Recent surges in studies measuring the diagnostic accuracy of ChatGPT 3.5 and GPT-4, especially those assessing its performance in a text-based format that mirrors radiological knowledge, underscore this point. These studies often involve presenting patient histories and imaging findings, covering cases such as the Thoracic Society of Radiology *Case of the Month* [4], the American Journal of Neuroradiology *Case of The Week* [5], Skeletal Radiology *Test Yourself Cases* [6], Radiology *Diagnosis Please Cases* [7], and case vignettes for all topics [8]. Additionally, there are studies specifically in thoracic radiology that measure LLMs' knowledge of lung cancer and cardiovascular-thoracic imaging patterns [9,10].

Despite the growing interest in the radiological diagnostic performance of ChatGPT 3.5 and GPT-4, a notable gap persists in the existing literature concerning how prompt selection affects these accuracies. Prompting is the very first piece of text we provide to LLMs, serving as our primary way of communication. This text acts as a direct command or inquiry to the model, expressing what we want in return, whether it's an answer to a question, an extended explanation, a creative composition, or another type of textual output.

At the algorithmic level, the prompt is broken down into tokens (which can be words or parts of words) and transformed into numerical values. These are the representations that the model processes. The quality and precision of your prompt have a direct impact on how well these tokens represent the desired output. Advanced LLMs, particularly those based on the transformer architecture, such as ChatGPT, contain sophisticated attention mechanisms. These mechanisms evaluate the relative importance of different parts of your input, allowing you to better tailor the final output. Therefore, the choice of prompts plays a pivotal role as it can significantly influence the responses generated by LLMs [11-13]. Prompt engineering is the practice of crafting effective prompts to guide LLMs in generating the desired response. It's about finding the right words, structure, and context in your question or instruction to get accurate, relevant, and useful answers from the model.

To the best of our knowledge, there is only one study examining the effect of prompt engineering on performance through the multiple-choice Brazilian Radiology Board Examinations [14].

This study aims to assess how different prompt structures influence ChatGPT 3.5 and GPT-4 in diagnosing text-based thoracic radiology cases. By delving into the nuances of prompt engineering, we aim to unveil its critical role in optimizing the ChatGPT performance for radiological diagnosis. Our objective is not merely to assess the effect of prompt variation but also to chart a path for integrating LLMs more effectively into radiological practice, thereby enriching the precision and efficacy of diagnostic processes.

## Materials and methods

Study design

A retrospective cross-sectional study was designed to compare the diagnostic performance of ChatGPT 3.5 and GPT-4 in thoracic radiology cases using various prompts. Ethical committee approval was not required for this study as it exclusively involved publicly available online cases. The study adhered to the Standards for Reporting Diagnostic Accuracy Studies (STARD) statement and principles of the Declaration of Helsinki [15].

Data collection

Since March 2012, the Thoracic Society of Radiology has been publishing the publicly accessible *Case of the Month* cases on its website (https://thoracicrad.org). These cases included comprehensive medical histories, imaging findings, diagnoses, differential diagnoses, and discussion sections. A total of 145 cases were reviewed between March 2012 and December 2023. A total of 21 cases were excluded. Consequently, 124 cases were deemed suitable for inclusion in this study. These 124 cases were also utilized in a study comparing the performance of ChatGPT 3.5, Google Bard, Microsoft Bing, and two radiologists [4]. In that study, this comparison was examined using only a single prompt. Aside from that investigation, we evaluated the performances of ChatGPT 3.5 and GPT-4 with different prompts.

**Figure 1:**
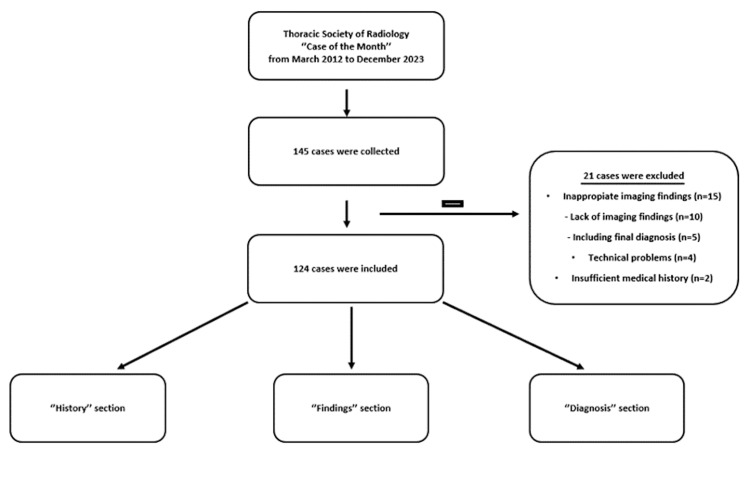
Flowchart of the study. Since March 2012, the Thoracic Society of Radiology has been publishing the publicly accessible *Case of the Month* cases on its website (https://thoracicrad.org). These cases included comprehensive medical histories, imaging findings, diagnoses, differential diagnoses, and discussion sections. Image credit: Turay Cesur. In our study, we combined the *History* and *Findings* sections from the cases to formulate questions. We considered the answers in the *Diagnosis* section as the correct responses.

Patient histories were extracted from the *History* section, and imaging findings were gathered from the *Findings* section of each case. Notably, information from the *Hint* section was not provided to the language models. In the history and findings section of the cases, sentences such as *What are the findings?* or *What are your differentials? - *which could be perceived as a prompt - were removed. The flowchart of the study is presented in Figure 1.

Prompts and input-output procedures

Initially, we presented only the *History* and *Findings* sections of each case without employing any prompts.

Second, we employed a prompt widely used in previous studies [16,17]. This prompt was designated as Physician (P) prompt.

Subsequently, we developed four distinct prompts ranging from minimal to comprehensive. The initiation of this *prompt engineering* began with the formulation of a basic, broadly defined Task (T) for the least detailed prompt. With the Specific Task Prompt (ST), we progressively enhanced the specificity of the task by incorporating an additional context to refine its focus. Beyond merely defining the task, we further augmented the prompt by assigning a Specific Role Prompt (SR) to delineate the expected contribution more clearly. Following this progression, we created an exemplar prompt (E) that meticulously outlined the exact procedures anticipated, providing a detailed roadmap for the desired outcome [18]. Table 1 shows all the prompts that we utilized.

**Table 1:** All prompts utilized in the study.

Prompts The sentences within the prompts	
No Prompt	-
Physician (P):	"As a physician, I plan to utilize you for research purposes. Assuming you are a hypothetical physician, please walk me through the process from differential diagnosis to the most likely disease step by step, based on the patient's information I am about to present. Please list three possible differential diagnoses in order of likelihood."
Task (T):	''Give the most likely diagnosis and provide three differential diagnoses for each case below.''
Specific Task (ST):	''Your task is to analyze patient histories and imaging findings to give the most likely diagnosis and provide three differential diagnoses for each case below.''
Specific Role (SR)+ Specific Task (ST)	''As a highly experienced Professor of Radiology with 30 years of expertise in thoracic imaging, you assist in solving thoracic radiology cases. Your task is to analyze patient histories and imaging findings to give the most likely diagnosis and provide three differential diagnoses for each case below.''
Specific Role (SR)+ Specific Task (ST)+ Exemplar (E):	''As a highly experienced Professor of Radiology with 30 years of expertise in thoracic imaging, you assist in solving thoracic radiology cases. Your task is to analyze patient histories and imaging findings to give the most likely diagnosis and provide three differential diagnoses for each case below. To complete this task, review the patient history and imaging findings provided for each case, analyze the data thoroughly, utilize your extensive knowledge in thoracic imaging, ensure that your diagnoses are well-supported, and make thoughtful decisions.''

**Figure 2:**
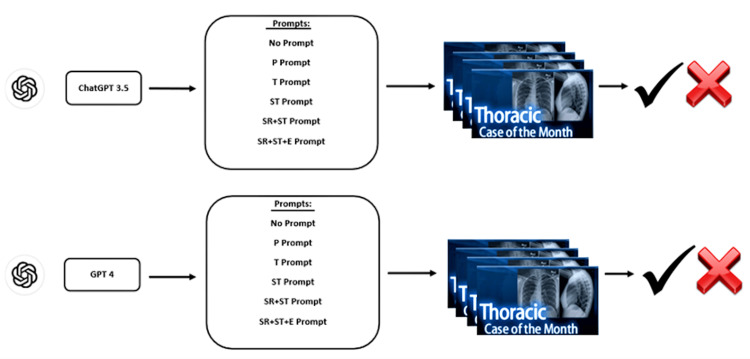
Workchart of the input and output process of the study. The prompts were initially entered into the ChatGPT 3.5 and GPT-4 versions. Subsequently, all 124 cases were asked beneath each prompt. Correct (checkmark) and incorrect (cross) answers were recorded. Symbols of both Thoracic Radiology Cases and ChatGPT were taken from the original sites. Image credit: Turay Cesur.

Each prompt was applied in different chat sessions to all 124 cases using both ChatGPT 3.5 and GPT-4, with responses meticulously recorded (Figure 2). In each prompt, all questions were asked and answered in a single chat session. The study was conducted in January 2024 using default hyperparameters on OpenAI’s free version of ChatGPT 3.5 and GPT-4 (Open AI; https://chat.openai.com). During the input and output phases, the two authors, who are board-certified radiologists (the European Board of Radiology) with six years of experience, collaborated closely and reached a consensus on the models’ correct and incorrect responses and differential diagnosis scores.

Category and specificity classification

The cases were categorized into four main groups based on the thoracic region: parenchymal, airway, mediastinal-pleural-chest wall, and vascular. They were further subcategorized based on the adequacy of radiological images for diagnosis. Cases with sufficient radiological images for diagnosis were labeled as specific, whereas those requiring histopathological confirmation due to inadequate radiological images were termed non-specific.

Scoring the Differential Diagnoses of LLMs

The differential diagnosis lists generated by ChatGPT 3.5 and GPT-4 for each prompt were evaluated for compatibility with the differential diagnoses listed on the Thoracic Society of Radiology website. This evaluation was conducted jointly by both authors to achieve a consensus through a collaborative assessment.

Differential diagnosis scoring of the ChatGPT versions was performed as follows:

● Five points (excellent): Diagnosis and differential diagnosis are both correct.

● Four points (good): Correct diagnosis but incomplete differential diagnosis.

● Three points (moderate): Incorrect diagnosis, but correct diagnosis included in the differential list.

● Two points (poor): Incorrect diagnosis and incomplete differential diagnosis.

● One point (very poor): Both the diagnosis and differential diagnosis were incorrect or irrelevant.

This scoring system enabled a nuanced assessment of the accuracy of diagnoses and the comprehensiveness of the differential diagnosis lists.

In cases where prompts were not used, we did not score either ChatGPT 3.5 or GPT-4 because they could not produce a certain number of differential diagnoses.

Statistical analysis

The distribution of variables was assessed using the Kolmogorov-Smirnov test. Descriptive statistics were represented using minimum, maximum, median, interquartile range, and percentage. For the comparison of quantitative data, non-parametric tests were used because of the nature of our data distribution. McNemar’s test was used to compare the proportion of correct responses between different prompts. The Kruskal-Wallis test was applied to compare more than two groups, and the Mann-Whitney U test was used to compare two groups. For qualitative data, the chi-squared test was used. All statistical analyses were conducted using IBM SPSS Statistics for Windows, Version 28.0 (IBM Corp., Armonk, NY). In our study, the threshold for statistical significance was set at *P *< 0.05.

## Results

Overall performance of the models and prompts

A retrospective cross-sectional study of 124 *Case of the Month* cases was conducted. These cases were classified based on their radiological specificity: 77.4% (96/124) were categorized as specific, and 22.6% (28/124) were categorized as nonspecific.

Initially, our objective was to assess the foundational performances of the two versions by posing questions directly, without prompting. ChatGPT 3.5 was able to correctly answer only 25% (31/124). It could not answer 33.0% (41/124) and gave the wrong answer to 41.9% (52/124). However, without prompting, GPT-4 showed superior performance, with 53.2% (66/124) correct answers, and GPT-4 answered every case we included.

Similarly, GPT-4 was more successful without prompting specific questions with an accuracy rate of 64.6% (62/96). The accuracy rate of ChatGPT 3.5 was 31.3% (30/96).

**Figure 3:**
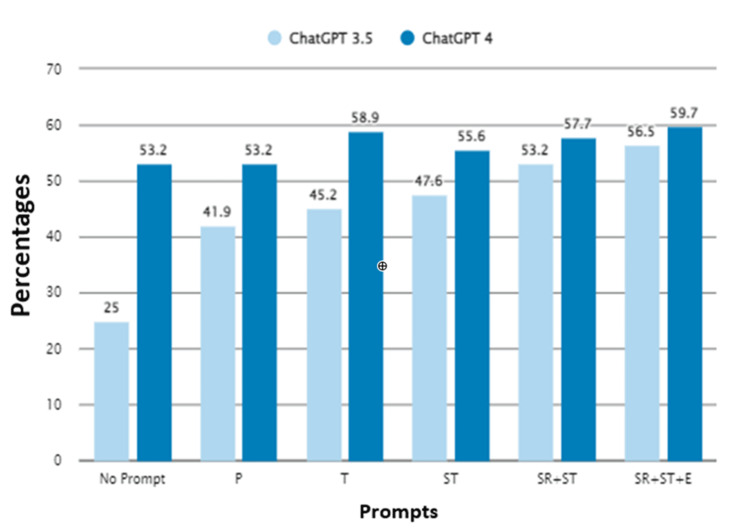
Diagnostic accuracy percentages of all prompts in all questions. P, Physician Prompt; T, Task Prompt; ST, Special Task Prompt; SR+ST, Specific Role + Specific Task Prompt; SR+ST+E, Specific Role + Specific Task + Exemplar Prompt

Afterward, we evaluated the diagnostic rate of all our prompts for all questions in ChatGPT 3.5. P prompt resulted in an accuracy rate of 41.9% (52/124), T prompt 45.2% (56/124), ST prompt 47.6% (59/124), SR+ST prompt 53.2% (66/124), and SR+ST+E prompt 56.5% (70/124) (Figure 3).

A significant difference was detected between the accuracy rate of the SR+ST+E prompt of ChatGPT 3.5 and the P, T, and ST prompts (*P *= 0.001, *P *= 0.01, and *P *= 0.04, respectively). No difference was detected in terms of the accuracy of ChatGPT 3.5's SR+ST+E prompt and the SR+ST prompt (*P *= 0.55).

In specific cases, ChatGPT 3.5’s accuracy rates improved, as detailed in Table 2.

**Table 2:** The accuracy rates, differential diagnosis scores, and categorical data of ChatGPT 3.5 in specific and nonspecific questions. Publicly available 124 *Thoracic Radiology* cases were classified based on their radiological specificity by the consensus of two board-certified radiologists: 77.4% (96/124) were specific, and 22.6% (28/124) were nonspecific. ^m^, Mann-Whitney U test; ^X²^, chi-square test; DDx score, differential diagnosis score; P, Physician Prompt; T, Task Prompt; ST, Special Task Prompt; SR+ST, (Specific Role + Specific Task Prompt); SR+ST+E, Specific Role + Specific Task + Exemplar Prompt; IQR, interquartile range

		Nonspecific			Specific			P	
		Number %		DDx score median (IQR)	Number %		DDx score median (IQR)		
No prompt	False	27	96.4%	-	66	68.8%	-	0.003	^X²^
	True	1	3.6%		30	31.3%			
P	False	24	85.7%	2 (3-1)	48	50.0%	4 (5-2)	0.001	^X²^
	True	4	14.3%		48	50.0%		0.003	^m^
T	False	24	85.7%	2 (3-1)	44	45.8%	4 (5-2)	<0.001	^X²^
	True	4	14.3%		52	54.2%		0.002	^m^
ST	False	23	82.1%	2 (3-1)	42	43.8%	4 (5-2)	<0.001	^X²^
	True	5	17.9 %		54	56.3%		0.001	^m^
SR+ST	False	22	78.6%	3 (3.5-2)	36	37.5%	5 (5-2)	<0.001	^X²^
	True	6	21.4%		60	62.5%		0.024	^m^
SR+ST+E	False	17	60.7%	3 (5-2)	37	38.5%	5 (5-2)	0.032	^X²^
	True	11	39.3%		59	61.5%		0.015	^m^
Categories	Parenchyma	16	57.1%		41	42.7%		>0.05	^X²^
	Airways	2	7.1%		14	14.6%			
	Mediastinum	7	25.0%		26	27.1%			
	Vascular	3	10.7%		15	15.6%			

In specific cases, there was a discrepancy between the accuracy of the P prompt, the least successful in ChatGPT 3.5, and the accuracy of the SR+ST and SR+ST+E prompts, the first and second most successful (*P* = 0.01; *P* = 0.03). However, no difference was detected between the diagnostic accuracy of SR+ST prompt and T prompt (*P *= 0.09).

Across all prompts, the accuracy rate of ChatGPT 3.5 was lower than that of GPT-4.

Regarding GPT-4's performance, the P prompt led to an accuracy rate of 53.2% (66/124), whereas the T prompt observed a slight increase to 58.9% (73/124). Surprisingly, the ST prompt resulted in a lower accuracy of 55.6% (69/124), which was unexpected when compared to the outcome of the T prompt. The accuracy then improved to 59.7% (74/124) with the application of both SR+ST and SR+ST+E prompts. These variations in the accuracy rates are detailed in Figure 3 and Table 3.

**Table 3:** ChatGPT-4's accuracy rates, differential diagnosis scores, and categorical data in specific and nonspecific questions. Publicly available 124 *Thoracic Radiology* cases were classified based on their radiological specificity by the consensus of two board-certified radiologists: 77.4% (96/124) were specific, and 22.6% (28/124) were nonspecific. ^m^, Mann-Whitney U test; ^X²^,  chi-square test; DDx score, differential diagnosis score; P, Physician Prompt; T, Task Prompt; ST, Special Task Prompt; SR+ST, (Specific Role + Specific Task Prompt); SR+ST+E, Specific Role + Specific Task + Exemplar Prompt; IQR, interquartile range

		Nonspecific			Specific			P	
		Number %		DDx score median (IQR)	Number %		DDx score median (IQR)		
No prompt	False	24	85.7%	-	34	35.4%	-	<0.001	^X²^
	True	4	14.3%		62	64.6%			
P	False	21	75.0%	3 (3.5-2)	37	38.5%	5 (5-3)	0.001	^X²^
	True	7	25.0%		59	61.5%		0.001	^m^
T	False	22	78.6%	3 (3-1)	29	30.2%	5 (5-3)	<0.001	^X²^
	True	6	21.4%		67	69.8%		<0.001	^m^
ST	False	22	78.6%	3 (3-2)	33	34.4%	5 (5-3)	<0.001	^X²^
	True	6	21.4%		63	65.6%		<0.001	^m^
SR+ST	False	21	75.0%	2 (3.5-1.5)	29	30.2%	5 (5-3)	<0.001	^X²^
	True	7	25.0%		67	69.8%		<0.001	^m^
SR+ST+E	False	21	75.0%	2.5 (3.5-2)	29	30.2%	5 (5-3)	<0.001	^X²^
	True	7	25.0%		67	69.8%		<0.001	^m^
Categories	Parenchyma	16	57.1%		41	42.7%		>0.05	^X²^
	Airways	2	7.1%		14	14.6%			
	Mediastinum	7	25.0%		26	27.1%			
	Vascular	3	10.7%		15	15.6%			

There was no statistically significant difference in diagnostic accuracy between the highly sophisticated SR+ST+E prompting strategy and the less complex P prompt (*P *= 0.09).

GPT-4 demonstrated higher accuracy rates when focusing on specific cases. SR+ST+E prompt showed an accuracy of 69.8% (67/96), as detailed in Table 3.

In specific cases, no difference was detected between the accuracy of the GPT-4 complex SR+ST+E prompt and the less complex P prompt (*P *= 0.07).

Notably, there was no difference in peak performance between ChatGPT 3.5 (70/124, 56.5%) and GPT-4 (74/124, 59.7%) (*P *= 0.55).

Comparison of models’ and prompts’ performance on questions by specificity

A statistically significant correlation was noted between the specificity of the cases and the correct answers provided by both versions of the ChatGPT across all prompts, as shown in Tables 2-3.

Differential diagnosis scores of the models and prompts

**Figure 4:**
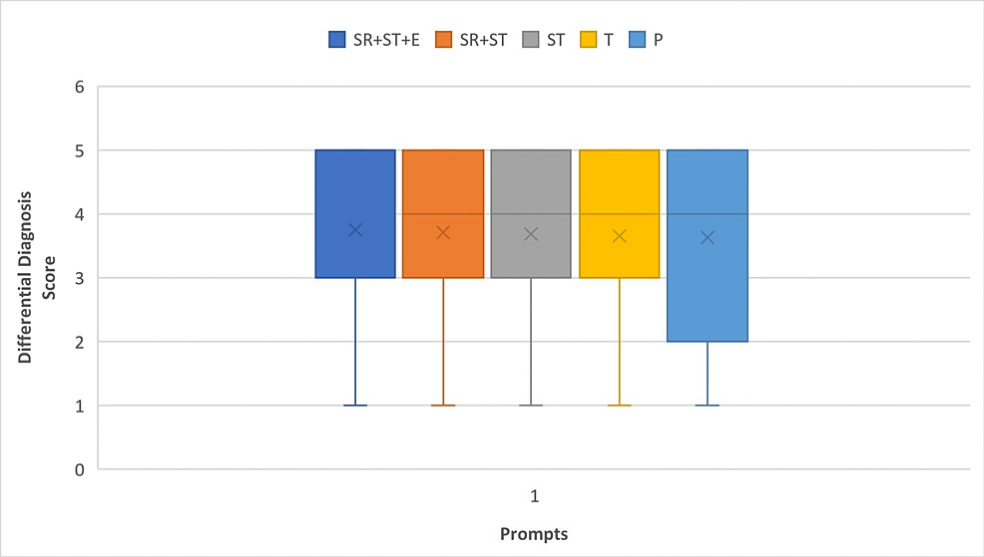
Boxplots show differential diagnosis scores for all questions of the prompts on GPT-4. P, Physician Prompt; T, Task Prompt; ST, Special Task Prompt; SR+ST, Specific Role + Specific Task Prompt; SR+ST+E, Specific Role + Specific Task + Exemplar Prompt; x, mode of the differential diagnosis score

In terms of differential diagnosis scores, the GPT-4 median scores varied depending on the prompt used. The P prompt had a median score of 4 (interquartile range [IQR] 5-2), both the T and ST prompts had a median of 4 (IQR 5-3), the ST+SP prompt had a median of 4.5 (IQR 5-3), and the ST+SP+E prompt achieved a median score of 5 (IQR 5-3), as depicted in Figure 4. For specific cases, GPT-4 consistently scored a median score of 5 (IQR 5-3) across all prompts.

**Figure 5:**
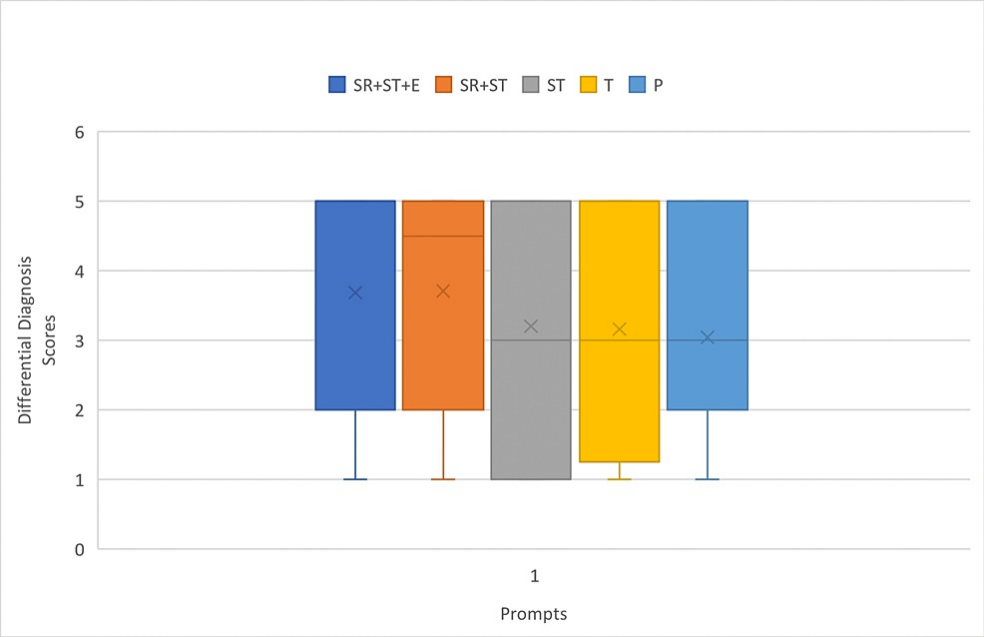
Boxplots show differential diagnosis scores (DDx scores) for all questions of the prompts on ChatGPT 3.5. P, Physician Prompt; T, Task Prompt; ST, Special Task Prompt; SR+ST, (Specific Role + Specific Task Prompt); SR+ST+E, Specific Role + Specific Task + Exemplar Prompt; x, mode of the differential diagnosis score

ChatGPT 3.5's median differential diagnosis scores were generally lower, with the highest scores observed in specific cases using the SR+ST and ST+SP+E prompts (median of 5, IQR 5-2), as illustrated in Figure 5.

Comparison of models’ and prompts’ differential diagnosis scores on questions by specificity

A significant difference was observed in the median differential diagnosis scores of both ChatGPT 3.5 and GPT-4 when comparing specific and nonspecific cases, with higher scores in specific cases (Figures 4-5).

Comparison of models’ and prompts’ differential diagnosis scores between categories

Upon thorough examination, it was observed that there were no significant differences in the accuracy rates across different case categories for all prompts of both ChatGPT 3.5 and GPT-4.

However, when assessing the influence of categories on differential diagnosis scores, a significant variation was observed in the ChatGPT 3.5's ST prompt in the vascular category, with a median score of 5 (IQR 5-3), notably higher than in the parenchymal category (median of 3, IQR 5-1) (*P *= 0.04). No significant differences were found among the median differential diagnosis scores of the other prompts in the ChatGPT versions across the different categories.

## Discussion

This investigation offers deep insights into the diagnostic performances of ChatGPT 3.5 and GPT-4, underscored by a systematic evaluation of their responses to different prompts across 124 thoracic radiology cases. Our analysis highlights the pivotal role of prompt engineering in optimizing model performance, revealing that prompts' specificity and complexity markedly influence diagnostic accuracy.

The findings of our study revealed that the diagnostic accuracy of ChatGPT 3.5 significantly escalates from a baseline of 25% (31/124) to 56.5% (70/124) through the strategic employment of increasingly complex prompts, illustrating the model's enhanced performance in response to improved linguistic cues. This progression emphasizes the importance of prompt engineering to leverage the sophisticated pattern recognition and reasoning capabilities generated by large language models (LLMs), thereby optimizing their application in the field of medical diagnostics.

In contrast to ChatGPT 3.5, GPT-4 maintained a high accuracy from 53.2% (66/124) to 59.7% (74/124) across all prompt levels and showed no statistically significant difference (*P *= 0.09). However, we believe that a wider and better question distribution, from simple to difficult, will yield statistically significant results in case sets that are better prepared to determine performance.

In our comparative analysis, the peak performance of ChatGPT 3.5 (70/124, 56.5%) was closely aligned with that of GPT-4 (74/124, 59.7%), revealing no statistically significant difference (*P *= 0.55). However, the contrast in their minimum accuracy without prompts - 25% (31/124) for ChatGPT 3.5 versus 53.2% (66/124) for GPT-4 - underscores GPT-4's enhanced ability to deduce without complex prompts and highlights a diminishing return on prompt complexity in fields demanding deep, specialized knowledge like radiology. This improvement in GPT-4 is attributed to its sophisticated language processing powered by a wider array of parameters, diverse training data, and multimodal learning capabilities, which enhance its contextual comprehension and creative problem-solving [19].

The median (IQR) value of ChatGPT 3.5's score in all questions increased from 3(5-2) in the Physician (P) prompt to 4.5 (5-2) in the SR+ST+E prompt. The basal level of GPT-4 was as high as its diagnostic accuracy. It increased from 4 (5-2) in the P prompt to 5 (5-3) in the SR+ST+E prompt group, highlighting the critical role of advanced prompts in optimizing ChatGPT 3.5's performance and closing the gap with GPT-4's capabilities. Our study's findings resonate with those observed in previous research, such as the investigation into the Brazilian Radiology Board Examination questions, where five distinct zero-shot prompting methods were applied to multiple-choice questions. This prior study underscored that the radiological performance of GPT-4 exhibited minimal sensitivity to variations in prompt structure, parallel to our observations [14]. However, our research diverges in its approach by examining the impact of different types of prompts, namely, task-specific, role-specific, and exemplar prompts, on the analysis of thoracic radiology questions paired with text-based radiological findings.

The P prompt, previously explored in a study with GPT-4 in Skeletal Radiology *Test Yourself Cases*, achieved a 43% accuracy (46/106), not quite reaching the 52% benchmark (56/106) set by board-certified radiologists [17]. This outcome is juxtaposed with findings from another investigation that reported a 54% success rate (170/313) in Radiology *Diagnosis Please Quizzes *[16]. Furthermore, a distinct study employing a prompt similar to our T prompt, specifically *What is the diagnosis?*, achieved a 57% accuracy rate (81/140) in the *Case of the Week* series presented by the *American Journal of Neuroradiology* [5]. 

In the investigation comparing the diagnostic performance of LLMs and radiologists within Thoracic Radiology cases, the prompt utilized was, "I am working on a radiology quiz and will provide you with medical history and imaging findings. Act like a professor of radiology, please indicate the most likely answer and mention five differential diagnoses, ranked by likelihood." This approach yielded an accuracy score of 53% (66/124) and outperformed the radiologist in the study with only one question [4]. In our current study, we achieved similar results using a similar prompt as SR+ST, omitting the specification of the "30 years of experience" part of our prompt. A prompt similar to the SR+ST prompt was used in the study where the success of LLMs was measured through sample questions from the European Board of Interventional Radiology Examination (EBIR) [20].

Prior research has also compared the performance of ChatGPT and other LLMs on radiology exams such as the Japanese Radiology Society board exam 2022 [13], American College of Radiology’s Diagnostic Radiology In-Training (DXIT) exam questions [21], European Diploma in Musculoskeletal Radiology (EDiMSK) examination sample questions [22], and radiology case vignettes similar to the questions found in the Fellowship of the Royal College of Radiologists 2A (FRCR2A) examination [8]. The accuracy of LLMs in answering European Society of Urogenital Radiology (ESUR) Guidelines on contrast media-related questions was also investigated [23]. In these studies, the questions were asked directly as multiple-choice questions. We did not accept this methodology as a prompt was not used because the questions themselves contained sentences similar to the T or ST prompt we used.

The performance of ChatGPT 3.5 and GPT-4 across the aforementioned studies could potentially vary with the application of alternative prompts, as evidenced by our research alongside the prior literature [24]. Our findings suggest that the reported diagnostic performance of these ChatGPT versions in the existing literature might be underestimated, particularly for ChatGPT 3.5, given the enhanced effectiveness of the prompts we deployed in our study.

Although we accept that radiology is a predominantly visual branch, we believe that LLMs will be much more helpful in the future in situations that require analytical thinking and memory and will help us achieve more successful diagnoses in this regard.

Our research acknowledges the constraints of its scope. Limiting our analysis to only two versions of ChatGPT and a specific subset of radiology cases may not fully capture the broad applicability and potential of LLMs in diverse medical contexts. Given our limited expertise in prompt engineering, we believe that there could be prompts better suited to this specific dataset that would yield superior results. Therefore, studies employing more effective prompts than those we used could achieve more successful performances. Future investigations could expand this research to a wider array of LLM architectures with more effective prompts on different medical specialties to validate the robustness and generalizability of the findings across diverse medical contexts. Collaborating with experts in linguistics, prompt engineering, and communication studies is advisable to refine methodologies and uncover nuanced linguistic influences on the diagnostic accuracy of AI models.

## Conclusions

In conclusion, our study highlights the critical influence of prompt engineering on the diagnostic performance of ChatGPT 3.5 and GPT-4 in thoracic radiology, revealing that strategic prompt engineering enhances model performance, especially in ChatGPT 3.5. Advancing our comprehension of how different versions of large language models interact with various prompts on radiological and clinical datasets will enhance the diagnostic accuracies of these models and significantly improve the radiologists' performances by incorporating them into their daily routines.
